# Adherence to face-down and non-supine positioning after macular hole surgery

**DOI:** 10.1186/s12886-018-0979-8

**Published:** 2018-12-14

**Authors:** Emi Morimoto, Yoshiaki Shimada, Mitsuo Sugimoto, Tadashi Mizuguchi, Atsuhiro Tanikawa, Masayuki Horiguchi

**Affiliations:** Department of Ophthalmology, Fujita Health University School of Medicine, 1-98 Dengakugakubo, Kutsukake-cho, Toyoake, Aichi 470-1192 Japan

**Keywords:** Adherence. Face-down positioning. Gas tamponade. Macular hole. Non-supine position. Vitrectomy

## Abstract

**Background:**

This study aimed to investigate patient adherence to face-down positioning (FDP) and non-supine positioning (NSP) following vitrectomy with gas tamponade for treating macular holes (MHs).

**Methods:**

Nursing records of 92 patients who underwent vitrectomy with gas tamponade for small-diameter (diameter < 400 μm) MHs during April 2016–June 2017 were examined. Forty-seven and 45 patients were instructed to maintain FDP and NSP (FDP and NSP groups), respectively. Patient adherence was evaluated seven times a day for 3 days, and the adherence rate was calculated.

**Results:**

The mean adherence rate was significantly higher in the NSP group (99.3% ± 2.7%) than in the FDP group (93.7% ± 13.3%; *P* < 0.001, Mann–Whitney U test). Forty-one patients (91.1%) in the NSP group had an adherence rate of 100%, which was significantly higher than that in the 24 patients in the FDP group (51.1%; *P* < 0.001, chi-squared test). No statistically significant difference was observed between the patients in the two groups regarding sex, age, MH diameter, and pre- and postoperative visual acuities. MH closure was achieved in all patients.

**Conclusions:**

Almost half of the patients in the FDP group did not obtain 100% adherence rate, suggesting that patient adherence was largely compromised. Patient adherence was better in the NSP group as patient compliance to NSP was better, however, 8.9% of patients were found in face-up positioning at least once. Incompleteness of patient adherence was common, although to differing degrees.

## Background

Face-down positioning (FDP) is the standard recovery posture following vitrectomy with gas tamponade for treating macular hole (MH) closure [1–40]. However, FDP is inconvenient and not easily tolerated by many patients, and thus, the duration of continuing FDP has been debated for years [2–8, 10–25, 29, 32–35]. Modifications in FDP to enhance tolerability, such as shortening the duration [2, 5, 8, 11, 12, 15, 17, 18, 23, 31, 32] and alleviated positioning, which generally avoids supine or face-up positioning (non-supine positioning, NSP) [6–8, 10–14, 16, 19, 21, 23–25, 29, 30, 34, 35] have previously been proposed. Prognoses observed after these modifications were statistically compared with those observed after strict adherence to FDP [5, 7, 8, 10–12, 22–25, 30]. However, these previous studies did not consider the influence of patient adherence. Assessments showed that adherence to FDP [4, 22, 26, 33, 37, 39] considerably varied among patients. Discussion about the requirement of FDP would be more meaningful if actual patient adherence is disclosed. Thus, this study aimed to investigate patient adherence to FDP and NSP for achieving MH closure.

## Methods

### Subjects

We retrospectively examined the nursing records of hospitalized patients who had undergone primary vitrectomy with gas tamponade at Fujita Health University Hospital (Toyoake, Japan) to treat idiopathic MHs of < 400 μm in diameter. From April 2016 to October 2016, patients were advised to maintain FDP for at least 3 days after the surgery (FDP group; 47 patients, 24 females and 23 males). From November 2016 to June 2017, patients were advised to maintain FDP for 3 h following surgery and to subsequently shift to NSP (NSP group; 45 patients, 26 females and 19 males). All patients were advised to maintain FDP or NSP as much as possible for at least 3 days. After MH closure was confirmed using optical coherence tomography, all patients including those in the FDP group were advised to maintain NSP until intraocular gas disappeared. All patients provided written, informed consent for surgery.

In the same period, patients with MHs ≥400 μm in diameter, which were thought to be difficult to close [29, 36] were treated with a different surgical technique using the inverted inner limiting membrane flap [9, 37]. To ensure the inversion of the ILM flap, the patients maintained in a sitting position immediately after the surgery for 3 h, and then in the FDP for 3 days [37]. Thus, the patients were excluded from this study.

### Surgery

Patients received instructions pertaining to FDP or NSP. All patients underwent a pars plana vitrectomy with triamcinolone-assisted internal limiting membrane peeling of approximately two disk diameters and gas tamponade with either 20% sulfur hexafluoride (SF_6_) or 15% perfluoropropane (C_3_F_8_). Additionally, prophylactic phacoemulsification and intraocular lens implantation were also performed in all 85 phakic patients (FDP group: 43 patients, NSP group: 42 patients).

### Nursing records

**Fig. 1:**
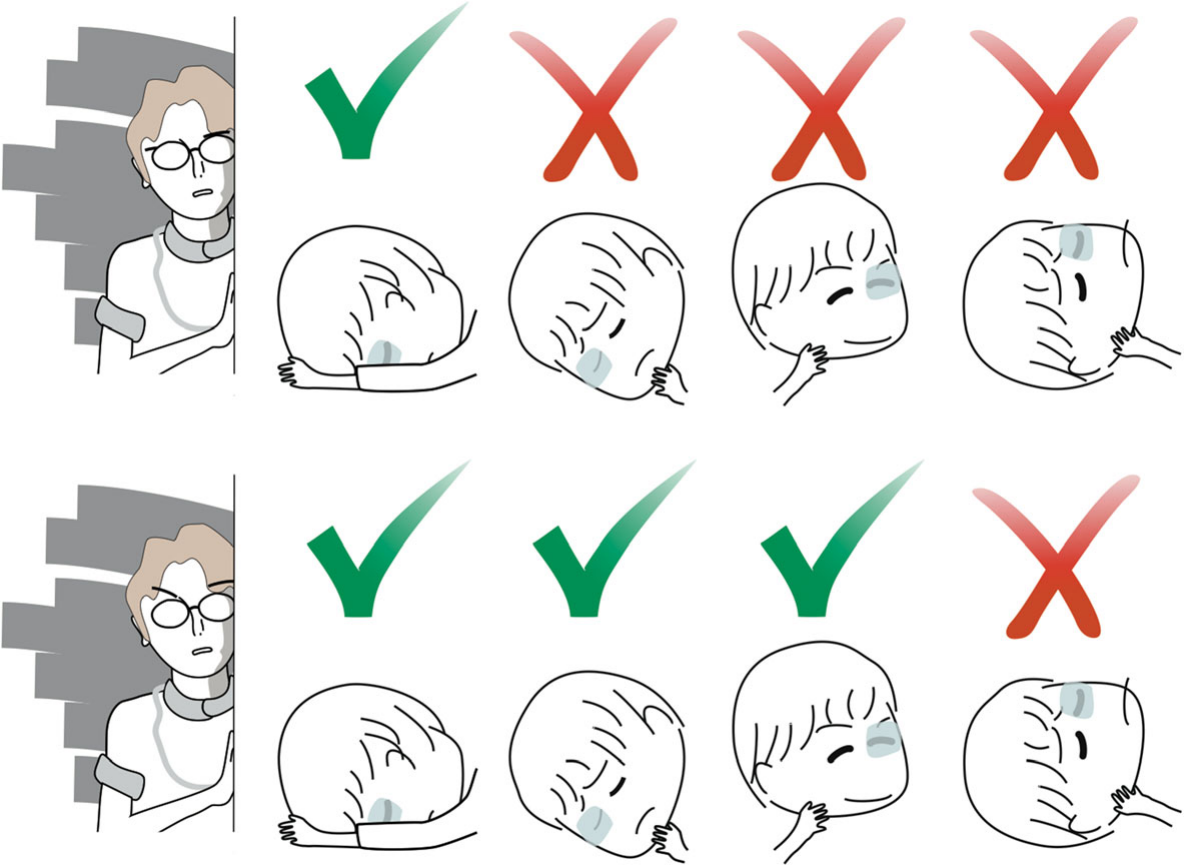
Observational adherence assessment. Patients were evaluated seven times a day, at 00:00, 03:00, 06:00, 10:00, 14:00, 19:00 and 21:00 for 3 days. In the FDP group (top panel), the patient passed the assessment only if the patient adhered to FDP. In the NSP group (lower panel), the patient failed only if the patient faced upward

Information regarding gas tamponade of patients was noted in hospital charts. Each time a patient was examined, the attending nurse recorded details regarding patient adherence to the recommended position (Fig. 1). In case of non-adherence, the nurse would instruct the patient to resume the recommended position and its maintenance even while sleeping.

Each time the patients were checked, the nurse recorded data on patient adherence into a handheld terminal. This data were then exported and stored in a digital hospital chart. In the FDP group, when the patient was not in the FDP, there were only occasional reports that the patient was often stayed in the supine position or sitting upright during the day hours, however, there was usually no further information on the body position.

### Adherence rate

To calculate patient adherence, the posture of each patient was checked seven times a day, at 00:00, 03:00, 06:00, 10:00, 14:00, 19:00 and 21:00 for 3 days. Patient monitoring began at 00:00 h immediately after the surgery. Although the nurses continued these evaluations until the gas disappeared or the patient was discharged, we included data obtained only from the first 3 postoperative days. While the 3-day assessment was also used in our previous studies, the observations in those studies were only four times a day [26, 38, 40]; this frequency was increased to 7 times a day in current study. Therefore, a total of 21 observations were recorded for each patient. The adherence rate was calculated as the percentage of the number of times the patient passed divided by the total number of observations. For example, if a patient was failed 3 of the 21 observations, the adherence rate was [(21–3)/21] × 100 = 85.7%.

## Results

Primary anatomical MH closure was achieved in all 92 patients (100%, Table 1).

**Table 1:** Patient demographics

	all subjects	FDP	NSP	P
N [cases]	92	47	45	
Eye [cases, right/male]	47/45	22/25	25/20	0.401 ^1)^
Gender [cases, female/male]	50/42	24/23	26/19	0.518 ^1)^
Age [years, mean + S.D]	65.6 + 7.8	65.2 + 9.2	66.0 + 6.0	0.620 ^2)^
MH diameter [*u*m, mean + S.D]	187.8 + 60.1	198.5 + 58.6	176.6 + 60.4	0.081 ^2)^
Adherence rate [%, mean + S.D]	96.4 + 10.0	93.7 + 13.3	99.3 + 2.7	< 0.001 ^3)^
A perfect 100% [cases]	65(70.7%)	24(51.1%)	41(91.1%)	< 0.001 ^1)^
Visual acuity [Log MAR, mean + S.D]				
Preoperative	0.52 + 0.28	0.52 + 0.26	0.52 + 0.30	0.548 ^2)^
Postoperative	0.13 + 0.20	0.10 + 0.15	0.16 + 0.24	0.139 ^2)^

1) Chi-squared test

2) Student *t* test

3) Mann-Whitney *U* test

**Fig. 2:**
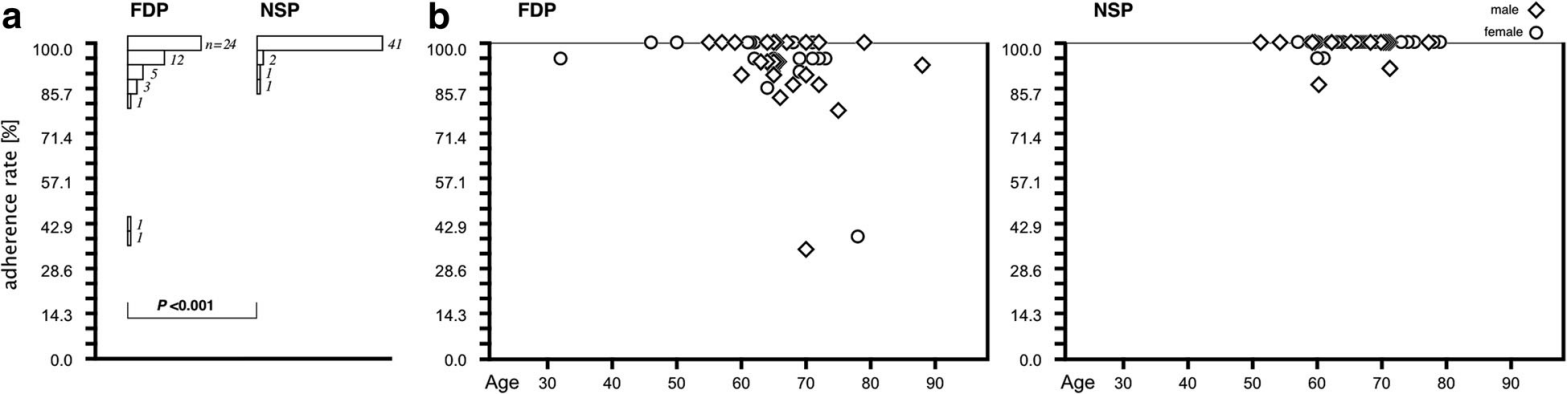
Adherence rate and distribution of adherence rates based on patient age. **a** Comparison of distribution of adherence rate in the FDP and NSP groups. **b** Distributions of the adherence rates based on patient age in the FDP and NSP groups

No significant difference were observed between the FDP and NSP groups regarding male: female, age, MH diameter, and pre- and postoperative visual acuities. However, MH diameter was smaller (*P* = 0.081) and postoperative visual acuity was lower (*P* = 0.139) in the NSP group than those in the FDP group, although these differences were not statistically significant. The mean adherence rate in the NSP group (99.3% ± 2.7%) was significantly higher than that in the FDP group (93.7% ± 13.3%; *P =* 2.30E-05, < 0.001, Mann–Whitney *U* test) (Fig. 2a).

The number of patients who had an adherence rate of 100% (*n* = 41, 91.1%) in the NSP group was significantly higher than that in the FDP group (*n* = 24; 51.1%; *P =* 2.48E-05, < 0.001, chi-squared test).

Figure 2b shows the distribution of the adherence rates based on patient age. No significant correlation was observed between the adherence rate and patient age.

Comparisons of adherence rates between the sexes are summarized in Table 2.

**Table 2:** Comparisons between the sexes

	FDP		P	NSP		P
	female	male		female	male	
N [cases]	24	23		26	19	
Adherence rate [%, mean + S.D]	94.6 + 14.1	92.8 + 14.1	0.457 ^1)^	99.6 + 1.3	98.7 + 3.8	0.695 ^1)^
A perfect 100% [cases]	13(54.2%)	11(47.8%)	0.664 ^2)^	24(92.3%)	17(89.5%)	0.714 ^2)^

1) Mann- Whitney *U* test

2) Chi-squared test

The mean adherence rates were, slightly higher in females than in males; however, they were not statistically significant (mean adherence rate in the FDP group, female: 94.6% ± 12.7%, male: 92.8% ± 14.1%, *P* = 0.457; in the FDP group, female: 99.6% ± 1.3%, male: 98.7% ± 3.8%, *P* = 0.695, Mann–Whitney *U* test).

## Discussion

### Assessment of adherence rate

Sensory devices have been developed and tested in pilot studies to assess the posture of the head [4, 10, 22, 33, 34, 39]. Although the such devices continuously record the position of the head for 24 h and its real-time feedback improves patient adherence [34, 39], it must be mounted on the patient’s head at all times, which can possibly increase the strain on patients. In this study, observational patient adherence assessment was based on a sampling frequency of only four [26, 38, 40] or seven times a day. However, patient adherence could be retrospectively obtained from nursing records of patients along with their surgical outcomes. As shown in our previous studies [26, 38, 40], adherence to FDP considerably varies among patients and is particularly higher in females than in males, although the differences between the sexes were not significant. Interestingly, patient age has little effect on the adherence rate [26, 38, 40].

### Adherence to NSP

It was not surprising that patient adherence to NSP was better than that to FDP considering better patient compliance to NSP [6–8, 10–14, 16, 19, 21, 23–25, 29, 30, 35, 36]. Almost half of the patients in the FDP group failed to obtain a 100% adherence rate and therefore the hypothesis that the patients would completely comply was largely compromised. Even if surgeons expect all their patients to always comply with the advice to continuously maintain FDP, some patients do not comply to these instructions. Despite of the incomplete adherence, MHs can be often closed, but the poor patient adherence to FDP can negatively impact the efficacy of the surgery [38]. In clinical trials, we speculate poor adherence to FDP may result in the underestimation of actual therapeutic effects of FDP that may be observed with strict adherence.

Advising patients to maintain NSP minimizes the gap between theoretical and actual practices. However, it should be also noted that 4 out of 45 (8.9%) patients in the NSP group were found in the face-up positioning at least once. The only thing they were advised to avoid was to lie in the face-up position. Despite these instructions, they did lie in the face-up position. Thus, incomplete patient adherence was common, although in varying degrees.

### FDP vs NSP

NSP easier to comply with and is thus beneficial for patients, resulting in good adherence. Moreover, MH closure was achieved in all patients with no proven adverse effects. Apparently, NSP was a favorable choice among patients. However, we cannot conclude that NSP is always better than FDP in postoperatively treating MHs. Our NSP protocol was protectively designed so as to minimize patient risk. Only MHs of < 400 μm in diameter were included. Patients were advised to maintain FDP for 3 h immediate after the surgery and to subsequently shift to NSP to facilitate MH closure. Although all MHs were closed, an inherent, unintentional limitation and insignificant differences, such as smaller MH diameter (*P* = 0.081) and lower postoperative visual acuity (*P* = 0.139), were observed in the NSP group compared with those in the FDP group. MH diameter is an important factor to be considered. Recent studies [29, 36] have reported that the combination of gas tamponade and NSP regimen achieved high closure rates for small/medium MHs (≤400 μm) but not for large-diameter MHs (> 400 μm). Further investigations to correlate surgical outcomes to NSP are warranted.

## Abbreviations

C_3_F_8_: Octafluoropropane
FDP: Face-down positioning
MH: Macular hole
NSP: Non-supine positioning
SF_6_: Sulfur hexafluoride
